# Factors Influencing the Time of Intubation Using C-MAC D-Blade® Video Laryngoscope: An Observational Cross-Sectional Study

**DOI:** 10.7759/cureus.34050

**Published:** 2023-01-22

**Authors:** Balasubramaniam Gayathri, Karthik Mani, Manoj Vishak, Joy John, Raghul G Srinivasan, Gunaseelan Mirunalini

**Affiliations:** 1 Anaesthesiology, SRM Medical College Hospital and Research Centre, Chennai, IND; 2 Anaesthesiology, Dr. Rela Institute & Medical Centre, Chennai, IND; 3 Centre for Statistics, SRM Institute of Science and Technology, Chennai, IND

**Keywords:** c-mac d-blade, video laryngoscope, intubation, resident, competency, curvature

## Abstract

Purpose

C-MAC D-Blade® (Karl Storz, Tuttlingen, Germany) video laryngoscope (VL) has proved to be of immense utility in difficult intubation. But unfortunately, in an urgent situation, the predictable correct curvature of the endotracheal tube for effortless intubation is not met. We hypothesized that expertise is the most important variable in intubation and that novice students will be unable to intubate if the angle of curvature is incorrect.

Methods

An observational cross-sectional study was planned with 30 anesthesia residents, categorized into three groups based on their expertise in laryngoscopy. Students had to intubate an airway mannequin using the C-MAC D-Blade® VL with three different stylet angulations. The curvatures were 80, 100, and 120 degrees, which are commonly encountered in routine day-to-day practice. The time to get a stable glottic view, time to intubate, and ease of intubation were measured.

Results

The mean time to intubate was the least with 100-degree angulation in group C (19.60 ± 0.97) while the maximum time was in group A with 80-degree angulation (61.49 ± 3.69). A significant difference was noted in time to get a stable glottic view when compared between the groups. There was no difference in time to intubate with different stylet angulations when compared between groups.

Conclusions

Novices and experts could intubate even if the angle of curvature was incorrect taking more time. The time to laryngoscopy is significantly dependent on experience, but the time to intubate is influenced by the angle of curvature of the stylet.

## Introduction

The C-MAC D-Blade® (Karl Storz, Tuttlingen, Germany) video laryngoscope (VL) is an intubation device that has made the management of the airway simpler [1]. VL has established itself as a better alternative for laryngoscopy when compared to standard Macintosh laryngoscopes [2]. C-MAC VL has also proved as an ideal laryngoscope for intubation training as the instructor can view the image on the monitor, assess the trainees’ performance, and guide them in real time. As the angle of the C-MAC D-Blade® VL is different from that of the endotracheal tube, it necessitates the use of a stylet to maneuver the endotracheal tube through the cords. Many times, this leads to increased intubation time even after getting a perfect glottic view, necessitating the use of a stylet to shape the tube [3-5]. Studies have already proved that the “hockey stick 90-degree angulation” is ideal for easy intubation while using a C-MAC D-Blade® [3]. But in practice, we often see that although instruction is given to technologists to angulate to 90 degrees, unfortunately in an urgent situation, the predictable correct curvature of the endotracheal tube for fast and easy intubation is not met. In effect, it has been seen that the angle of the tube ranges from 60 to 120 degrees as a result of over or under-bending the same. This can lead to delayed intubation time, desaturation, and failed intubation [6].

The study was performed by three groups of residents ranging from the ones with no expertise in performing video laryngoscopy to the ones with an acceptable level of expertise. The aim of the study was to find the most important variable influencing intubation between the competence gained with years of expertise and the angle of curvature of the stylet. We hypothesized that students who are not well versed in the technique of intubation using a VL will find it more difficult to intubate with inappropriate tube curvatures than the experts.

## Materials and methods

**Figure 1:**
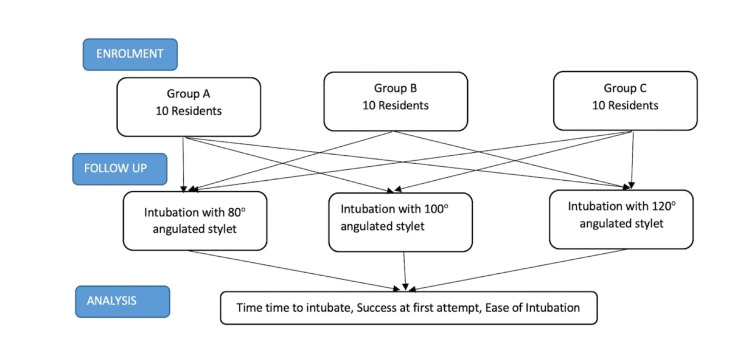
Group allocation and process flow

After obtaining the Institutional Ethics Committee's approval, the study was conducted on an airway simulation mannequin in the skill lab of a hospital in South India. The aim of the study was to find the time to get a stable glottic view, time to remove the stylet, intubation time, and ease of intubation using three different curvatures of stylet endotracheal tubes by students of three different competency levels. The study participants were residents of the department of anesthesiology in a medical college hospital in South India. Written informed consent for participation was obtained. Students of both genders from the first to the third year of postgraduation were included. The study was planned and executed as per the Strengthening the Reporting of Observational Studies in Epidemiology (STROBE) guidelines for observational studies. A total of 30 anesthesiology residents, 10 each for the first, second, and third year, enrolled for the study. Residents were categorized into three groups based on their prior experience in performing laryngoscopy: group A - no experience in performing C-MAC VL intubations; group B - have experience in performing less than or up to 10 C-MAC VL intubations; and group C - have experience of more than 10 C-MAC VL intubations. The three stylet tracheal tube conﬁgurations studied were 80-degree angulated stylet, 100-degree angulated stylet, and 120-degree angulated stylet (Figure 1).

The angle was created at about 8 cm from the distal tip of the 7.0 size cuffed endotracheal tube. Residents in each group were given a standardized live demonstration on how to intubate using the C-MAC VL with size 4 D-Blade. Residents were allowed one practice C-MAC VL intubation with each stylet configuration on the airway mannequin with an anatomically normal airway before the actual session. Residents were given equal chance to intubate with all three different stylet configurations.

Intubations were done with a 7.0-mm cuffed tracheal tube. In cases where difficulty was encountered, external laryngeal manipulation was applied. Once the confirmation of successful intubation was made, the stylet was removed. The endpoints were (a) stable glottic view, which was defined as the time taken to get a “Cormack-Lehane 4” view of the larynx; (b) time to intubate, which was defined as the time taken for the successful pass of the tube through cords; and (c) time to remove the stylet. A failed intubation attempt was deﬁned as an “inability to intubate the trachea within 60 seconds with a maximum of three attempts.” Participants were asked to score the ease of tracheal intubation on a six-point Likert scale with a score of “1” for very difficult intubation to “6” for very easy intubation.

Results were analyzed using SPSS 2015 trial version software (IBM Corp., Armonk, NY). Descriptive statistics included in the study were mean, standard deviation, and frequency. The Kolmogorov-Smirnov test used to determine the normalcy of variables showed p < 0.05 indicating variables did not follow a normal pattern. Consequently, analysis of various time points between the groups and angulations was done using the non-parametric Kruskal-Wallis test. The results were expressed at a 95% confidence interval. A p-value of <0.05 was considered statistically significant.

## Results

Time to get a stable glottic view

A significant difference was noticed in the time taken to get a stable glottic view between groups A, B, and C (p < 0.05). But on analyzing the influence of angulation as a variable, we noted no significant difference in the time to get a stable glottic view using three different angulations (p = 0.685; Table 1).

**Table 1:** Analysis of various variables influencing time taken during the process of intubation * Mean time of all the intubations in groups A, B, and C, respectively. ** Mean time of all the intubations using 80, 100, and 120-degree angulations, respectively.

	Group*	N	Mean	P-value	Angulation**	N	Mean	P-value
Time to stable glottic view	Group A	30	69.53		80°	30	48.5	
	Group B	30	37.4		100°	30	43.93	
	Group C	30	29.57	0.001	120°	30	44.07	0.685
Time to intubate	Group	N	Mean		Angulation	N	Mean	
	Group A	30	50.58		80°	30	75.5	
	Group B	30	44.43		100°	30	25.98	
	Group C	30	41.48	0.385	120°	30	35.02	0.001
Time to remove the stylet	Group	N	Mean		Angulation	N	Mean	
	Group A	30	43.37		80°	30	49.37	
	Group B	30	42.13		100°	30	43.87	
	Group C	30	42.76	0.324	120°	30	43.27	0.576

Time to remove the stylet

It was noticed that the stylet could be removed after intubation at about the same time in 80, 100, and 120-degree angulations, with no significant difference noted between the groups (p = 0.324). When angulation of the stylet of the variable was analyzed, again no difference was found (Table 1).

Time to intubation

When compared between groups A, B, and C, a significant difference was found between 80, 100, and 120-degree angulations when analyzed.

**Figure 2:**
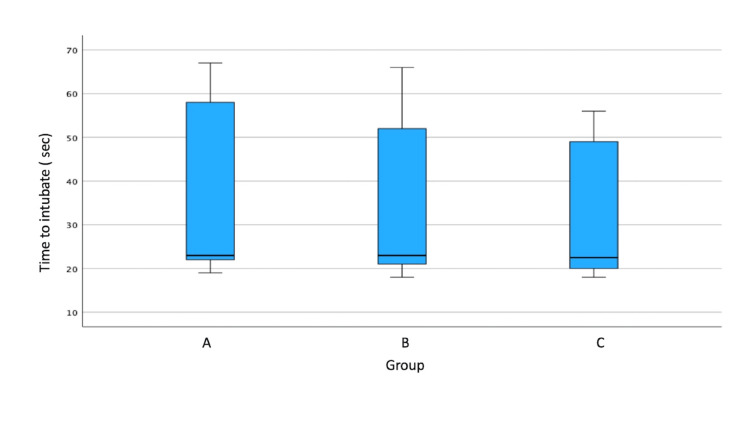
Time to intubate

On analyzing the results, we found no significant difference in the time to intubate between groups A, B, and C (p = 0.385). But a significant difference was found in the time taken to intubate using the three stylet curvatures (80, 100, and 120 degrees; p < 0.001; Table 1). Among all the groups, the minimal mean time for intubation was observed in group C with 100-degree angulation (19.60 ± 0.97), while the maximum time was observed in group A with 80-degree angulation (61.49 ± 3.69; Figure 2).

Ease of intubation

Students, irrespective of the group, gave a score of <3 (difficult to intubate) for intubation using 80-degree angulation. Likewise, students, irrespective of the group, gave a score of >3 (easy to intubate) with intubation using 100-degree angulation. A significant difference was found in the Likert score given by students for intubation using 120-degree angulation, as shown by a significant p-value of <0.05 in the Kruskal-Wallis test (Table 2).

**Table 2:** Ease of intubation

	Subgroup angulation	Likert < 3	Likert > 3	P-value
Group A	80 degrees	30	0	
	100 degrees	0	30	
	120 degrees	15	15	
Group B	80 degrees	30	0	<0.05
	100 degrees	0	30	
	120 degrees	4	26	
Group C	80 degrees	30	0	
	100 degrees	0	30	
	120 degrees	1	29	

## Discussion

Tracheal intubation remains a significant life-saving intervention in the field of anesthesia and emergency medicine. The reported incidence of difficult intubation ranges from 0.4% to 4.7%; rising to 5.7% in obstetric cases and 13.3% in obese patients [7,8]. Direct laryngoscopy (DL) with intubation is reported to be impossible in 0.43% of the population needing to move ahead with a surgical airway. VL has established itself as a better alternative for laryngoscopy and intubation in skill training as well as in the clinical context. Multiple studies have proved that mastering VL has a positive impact on the competency level of novices with skills transferable to DL [8,9]. Kılıçaslan et al. compared VL with a traditional Macintosh blade and found that the D-Blade especially reduces pressure on the teeth and shows better Cormack-Lehane views [10]. Studies have already proved that the “hockey stick 90-degree angulation” is ideal for easy intubation while using a C-MAC D-Blade® [3]. Simulation of endotracheal intubation in an anatomically correct mannequin is being used as a reliable surrogate for clinical context. Airway simulators are quite realistic in creating the feel and subtle aspects of human anatomy. As difficult intubation is fraught with ethical problems, training and evaluation in a mannequin work out as a doable alternative to train students in laryngoscopy [9-11]. We designed this study to find whether it is the competence gained with practice and performance over years or is it the angle of curvature of the stylet that is the most important variable influencing effortless intubation in a normal airway mannequin in a simulation lab.

To remove the influence due to the unfamiliarity with the anatomy of the mannequin, we incorporated one practice session with all the different angulations. All participants were aware and acquainted with the use of the most commonly used standard, i.e., the Macintosh laryngoscope. Once the participants had familiarized themselves with the mannequin, it was accepted that there should be no learning curve in using the C-MAC laryngoscope for intubation.

In our observation, we found that students in group A took a significantly longer time to get a stable laryngoscopic view when compared to the other groups. Students in group A had no previous experience with intubation using VL. Amalric et al., using multivariate analysis, evaluated the intubation success rate on the first attempt in the ICU after specific VL skill training [12]. They reported that the number of previous VL performed is an independent factor of first-attempt success at intubation.

Students in group A were novices in intubation using VL and took 69.53 ± 9.76 seconds to get a stable glottic view to intubate, while students from group C, who had done more than 10 intubations, took 29.57 ± 5.34 seconds. Time to intubate with 80-degree angulation was highest in group A, who were novices, and least in group C, who had expertise in intubation. The mean intubation time in group A using 80-degree stylet angulation was 61.49 ± 3.69, which is quite long and clinically relevant. It is important to note that the mean time of all the intubations from the three groups using 80-degree stylet was 75.5 ± 8.89, which is the longest and clinically significant. The mean time of all the intubations from the three groups using 100-degree stylet was 25.98 ± 8.89, which is the least and acceptable. This indicates that the most important parameter in learning laryngoscopy is to get a good glottic view. But when compared with different stylet angles, we found a definite increase in the time taken to intubate using 80-degree angulation of the stylet. This implies that irrespective of expertise, if angulation is incorrect (80 or 120 degrees), intubation will be difficult. This reiterates the point that the correct technique should be used for fast and successful intubation.

Buis et al., in their systematic review on defining the learning curve for endotracheal intubation, show that at least 50 endotracheal intubations are needed to be performed in elective circumstances to reach a minimum success rate of 90%. But on the contrary, VL has a short steep learning curve and the operators mastered the technique with just three practice attempts. The skills learned were retained for up to 12 weeks and there was no increase in intubation time even after a gap [13]. Sakles et al. did a retrospective analysis of intubations performed by residents in the emergency department [14]. They demonstrated that there was no significant improvement in DL between postgraduate year (PGY)-1 and PGY-3 residents. On the contrary, they noticed a considerable improvement in performance with VL between PGY-1 and PGY-3 residents. They observed that the learning curve for DL is fairly flat, with little or no improvement in success with the training. On the other hand, they observed that the VL has a steep learning curve with improvement in skills noticed over the course of residency training [14]. Whether DL or VL, operator skills do matter [15].

C-MAC VL is a second-line instrument, a gadget used in cases where the standard Macintosh laryngoscope fails. Getting a good view of the larynx with a D-Blade is quite easy, but to maneuver the tube through the glottis, we need proper angulation of the tube to 90-100 degrees with a stylet. In cases of anticipated difficult intubation, VL should perhaps be considered a first-line intubation strategy.

Limitations

The study was conducted with a small cohort of students in a mannequin with normal airways. A large-scale study with more participants will normalize the results and help to re-analyze the important factors resulting in successful intubation.

## Conclusions

VL can be learned easily by students. The ideal angle for the stylet should be 90-100 degrees. Even after mastering the art of laryngoscopy, if the tube with proper angulation is not provided, the laryngoscopist falters and takes a long time to intubate or may fail totally. The time to laryngoscopy is significantly dependent on training or the number of previous intubations, but the time to intubate is influenced by the angle of curvature of the stylet.
